# *In Vitro* Cytoprotective Effects and Antioxidant Capacity of Phenolic Compounds from the Leaves of *Swietenia macrophylla*

**DOI:** 10.3390/molecules201018777

**Published:** 2015-10-16

**Authors:** Sônia Pamplona, Paulo Sá, Dielly Lopes, Edmar Costa, Elizabeth Yamada, Consuelo e Silva, Mara Arruda, Jesus Souza, Milton da Silva

**Affiliations:** 1 Programa de Pós-Graduação em Química, Instituto de Ciências Exatas e Naturais, Campus Universitário do Guamá, Universidade Federal do Pará, Belém-PA 66075-970, Brazil; E-Mails: sgpamplona@yahoo.com.br (S.P.); paulorcsa@gmail.com (P.S.); yumikoyoshioka@yahoo.com.br (C.S.); msparruda@gmail.com (M.A.); 2 Laboratório de Neuropatologia Experimental (LaNEx), Hospital Universitário Barros Barreto, Rua dos Mundurucus, 4487–Guamá. CEP 66073-000. Belém, PA, Brazil; E-Mails: dicatrina@gmail.com (D.L.); etcosta@globo.com (E.C.); esyamada2013@gmail.com (E.Y.); 3 Faculdade de Engenharia de Alimentos, Instituto de Tecnologia, Campus Universitário do Guamá, Universidade Federal do Pará, Belém-PA 66075-970, Brazil; E-Mail: jesussouza@yahoo.com.br

**Keywords:** *Swietenia macrophylla*, cytoprotection, antioxidant capacity, polyphenols, LC-MS/MS

## Abstract

*Swietenia macrophylla* (mahogany) is a highly valued timber species, whereas the leaves are considered to be waste product. A total of 27 phenolic compounds were identified in aqueous extracts from mahogany leaves by comparing retention times and mass spectra data with those of authentic standards using LC-ESI-MS/MS. Polyphenols play an important role in plants as defense mechanisms against pests and pathogens and have potent antioxidant properties. In terms of health applications, interest has increased considerably in naturally occurring antioxidant sources, since they can retard the progress of many important neurodegenerative diseases such as Alzheimer’s and Parkinson’s diseases. The antioxidant capacities of two aqueous extracts, M1 (decoction) and M2 (infusion), were measured using TEAC and Folin-Ciocalteau methods. Additionally, M1 was used in order to investigate its potential cytoprotective effects on an *in vitro* model of neurodegeneration, by using primary cerebellar cultures exposed to methyl mercury (MeHg). Under experimental sub-chronic conditions (72 h), concomitant exposure of the same cultures to MeHg and M1 extract resulted in a statistically significant increase in cell viability in all three concentrations tested (10, 50 and 100 μg/mL), strongly suggesting that due to its high content of antioxidant compounds, the M1 extract provides significant cytoprotection against the MeHg-induced in vitro neurotoxicity.

## 1. Introduction

Serious pathologies such as cancer, senescence and inflammation, harm the membranes of biological systems causing overproduction of radical reactive species and leading to lipid peroxidation [1]. Although free radicals are known to play a physiological role in optimal cell function, excessive oxidative stress has been implicated in a variety of neurodegenerative diseases, including Alzheimer’s disease, Parkinson’s disease and amyotrophic lateral sclerosis [2]. Oxidative stress also plays an important role in other degenerative conditions such as autoimmune and inflammatory diseases (*i.e.*, ischemia and rheumatoid arthritis), cancer, diabetes mellitus, and atherosclerosis [3], as well as in metal-induced toxicity [4]. It has been suggested that polyphenolic content has a relationship with free radical-scavenging ability, and it has thus received increasing attention over the last decade, especially due to its potential protective effects against degenerative diseases linked to oxidative stress [5,6].

*Swietenia macrophylla*, commonly known as mahogany, belongs to the Meliaceae family and is distributed naturally from southern Mexico throughout Central and Tropical South America to Bolivia and Brazil, including large portions of the Amazon Basin [7,8]. It is one of the most valuable plants on the international market and because of its beauty and durability its wood is used to make products such as fine furniture and cabinetry, interior trim, paneling, fancy veneers, musical instruments, boat building, pattern making, turnery and carving [9].

Some studies have been reported in the literature about mahogany leaves, bark and seeds providing limonoids [10,11,12] and their derivatives. In a previous paper, we described the isolation and structural elucidation of six phragmalin-type limonoids from the hexane extract from the leaves of *S*. *macrophylla* [10]. Additionally, polyphenolic compounds such as flavonoids have been isolated from *Swietenia macrophylla* [9].

Falah *et al*. [9] reported potent antioxidant activity related to catechin and epicatechin isolated from *S. macrophylla* bark therefore, the study of *S. macrophylla* leaves, potentially rich in polyphenols, may lead to the discovery of new, useful antioxidant sources, providing an incentive for utilization of waste material from the timber industry and above all, the preservation of these plants.

HPLC coupled with an UV-Vis diode array detector (DAD) has been a method of choice for separating and isolating polyphenols [13,14]. However, identification of compounds from the patterns can be done safely using LC-HRESITOF-MS analysis.

Several methods can be used to measure the antioxidant capacity of plant extracts. Among these, the Trolox Equivalent Antioxidant Capacity (TEAC) assay and Folin-Ciocalteu assay are considered as reference methods [14]. The TEAC assay measures antioxidant inhibition based on the use of the relatively long-lived ABTS^•+^ by hydrogen atom transfer and thus reflects the classical radical chain breaking antioxidant activity [14,15]. The Folin-Ciocalteu assay, which is widely used to quantify the total phenolic content, can be considered as another antioxidant method since the mechanism involved is an electron transfer aiming at neutralizing an oxidant species [16].

Seeking to come up with an analytical method suitable for analyzing polyphenolic compounds in small concentrations in complex mixtures such as aqueous leaf extract, we have developed a LC-HRESITOF-MS procedure which is described herein. This method has proved to be feasible for the analysis of leaf infusions (aqueous extracts, popularly known as “tea”), and has also been applied to mahogany samples in order to illustrate the potential of this method for identification analyses.

LC-MS/MS analyses were also performed to confirm the presence of flavonoids and phenolic acids in aqueous extracts from leaves of *S*. *macrophylla*. The LC-MS/MS system is considered one of the most prominent methods in drug development using medicinal plants [17,18].

The aim of this work was to evaluate and compare the antioxidant activity of two aqueous extracts of *S. macrophylla* leaves—M1 prepared by decoction and M2 by infusion—as well as to identify some of their major polyphenolic compounds by using both the TEAC and the Folin-Ciocalteu assays. Additionally, in the context of the occurrence of oxidative stress in several conditions affecting the nervous system, we used one of these extracts (M1) in order to investigate its potential cytoprotective effects on an *in vitro* model of neurodegeneration, by using cerebellar primary cultures exposed to methyl mercury (MeHg), a neurotoxicant known to cause oxidative stress both in humans and in experimental models.

## 2. Results and Discussion

### 2.1. Antioxidant Assays-Trolox Equivalent Antioxidant Capacity (TEAC) and Folin-Ciocalteu Assays

The antioxidant capacities presented by two aqueous extracts of *S. macrophylla* are shown in Table 1. The values presented for both methods presented no significant differences between the forms of preparation of extracts (*p* < 0.05), which agrees with Almajano *et al.* [19] who evaluated the antioxidant capacity of different infusions of teas. The antioxidant capacity presented by the two methods shows that the leaf extract of *S. macrophylla* is an important antioxidant compound source when compared with other Amazonian plant leaf extracts [15].

**Table 1:** Folin-Ciocalteu (FC) and TEAC values of two aqueous extracts (M1 and M2) of *S. macrophylla* leaves. Values are expressed as Gallic acid equivalent (GAE) or mmol of Trolox equivalent (TE) both per g of dry crude extract (DCE).

Extracts	TP	TEAC
	mg GAE per g DCE	mmol TE per g DCE
M1	228.10 ± 2.40	2.43 ± 0.06
M2	245.15 ± 9.69	2.11 ± 0.14

### 2.2. Identification of Phenolic Compounds in Aqueous Extract of S. Macrophylla Leaves

**Figure 1:**
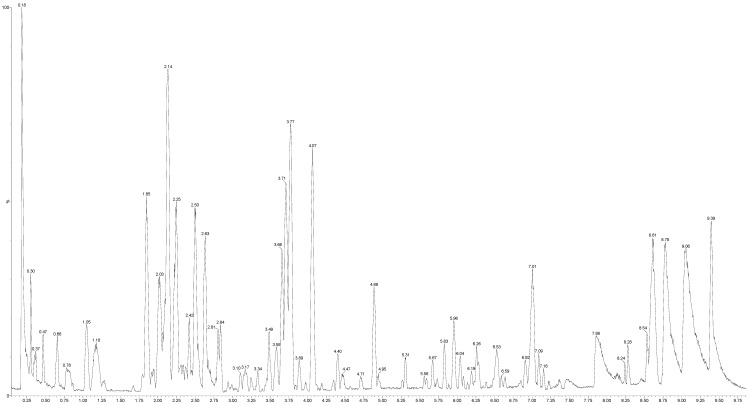
Total ion current chromatogram of aqueous extract of *S. macrophylla* leaves.

The M1 extract was eluted by gradient mode and the total ion current (TIC) chromatogram (Figure 1) was acquired by UPLC-TOF/MS, in which 27 phenolic compounds including phenolic acids and flavonoids have been identified. The mass spectrum of these compounds was obtained in positive ion mode by a MS^e^ experiment. This experiment makes it possible to observe in a single analysis both the molecular ion and the fragmentation of each identified component. The identification of polyphenols from M1 extract was carried out by comparing retention times and mass spectra with those of commercial standards using their respective *m*/*z* ratios.

The M1 extract showed a similar chromatographic profile when compared with standard compounds, whose peaks have been assigned in Table 2. All the standard compounds were detected and confirmed in the M1 extract.

**Table 2:** Phenolic compounds with their respective retention times and *m*/*z* ratio identified in the M1 extract.

Compounds	Empirical Formula	RT (min)	[M + H]^+^ Calcd	[M + H]^+^ obsd	Error (ppm)
Benzoic acid	C_7_H_5_O_2_	0.41	123.0446	123.0446	0.00
*p*-Hydroxybenzoic acid	C_7_H_6_O_3_	1.73	139.0395	139.0390	3.59
Cinnamic acid	C_9_H_8_O_2_	3.36	149.0602	149.0600	1.34
Gentisic acid	C_7_H_6_O_4_	1.26	155.0344	155.0343	1.29
Protocatechuic acid	C_7_H_6_O_4_	1.28	155.0344	155.0342	1.29
Synaptic acid	C_9_H_8_O_3_	2.22	165.0551	165.0548	1.81
*p*-Coumaric acid	C_9_H_7_O_3_	2.23	165.0551	165.0548	1.81
Gallic acid	C_7_H_5_O_5_	0.81	171.0293	171.0291	1.16
Syringic acid	C_9_H_9_O_5_	2.90	199.0606	199.0607	0.50
Resveratrol	C_14_H_12_O_3_	3.99	229.0864	229.0864	0.00
Chrysin	C_15_H_10_O_5_	1.16	271.0606	271.0611	1.84
Apigenin	C_15_H_9_O_5_	3.78	273.0762	273.0765	1.09
Naringenin	C_15_H_12_O_5_	3.88	273.0762	273.0766	1.46
Luteolin	C_15_H_9_O_6_	4.38	287.0555	287.0555	0.00
Kaempferol	C_15_H_10_O_6_	3.98	287.0555	287.0554	0.34
Epicatechin	C_15_H_14_O_6_	2.23	291.0868	291.0873	1.71
Catechin	C_15_H_14_O_6_	2.83	291.0868	291.0868	0.00
Quercetin	C_15_H_9_O_7_	3.76	303.0504	303.0506	0.66
Epigallocatechin	C_15_H_14_O_7_	1.07	307.0817	307.0824	2.27
Gallocatechin	C_15_H_13_O_7_	1.16	307.0817	307.0823	1.95
Myricetin	C_15_H_10_O_8_	2.38	319.0822	319.0822	0.00
Apigenin-7-*O*-glucoside	C_21_H_21_O_10_	3.84	435.1291	435.1289	0.45
Kaempferol-3-*O*-glucoside	C_21_H_20_O_11_	4.06	449.1083	449.1077	1.33
Quercetin-3-*O*-rhaminoside	C_21_H_19_O_12_	3.71	465.1033	465.1027	1.29
Quercetin-3-*O*-glucoside	C_21_H_19_O_12_	3.76	465.1033	465.1028	1.07
Kaempferol-3-*O*-rutinoside	C_27_H_30_O_15_	4.06	595.1662	595.1660	0.35
Quercetin-3-*O*-rutinoside	C_27_H_30_O_16_	3.76	611.1612	611.1609	0.49

Identification confirmed using commercial standards.

### 2.3. Effects of M1 Extract on Cell Viability of Cerebellar Primary Cultures

Based on chromatographic profiles of the two extracts M1 and M2, obtained by LC-UV and antioxidant capacity data, it was possible to see that the results showed no significant differences. Thus, cytoprotective tests were conducted only with the extract obtained by decoction (M1), since this is the most widely used form for preparing medicinal plants in the region, and also produced the highest yield.

**Figure 2:**
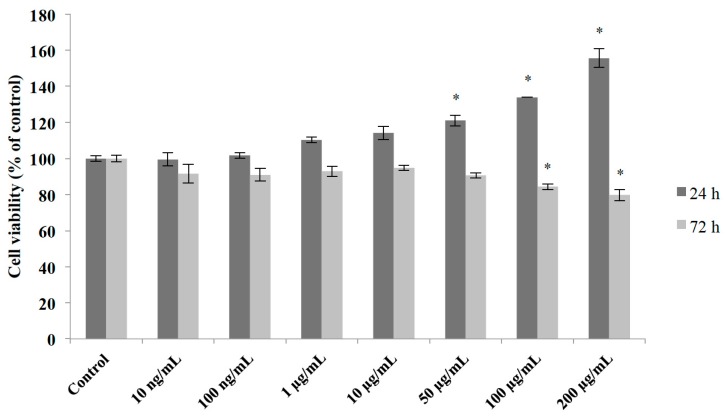
Effects of different dilutions of an aqueous extract of mahogany leaves (M1) on cell viability of cerebellar cultures. Cell viability was determined by MTT assay and is expressed as the percentage of untreated controls; mean + S.E.M. values of three-four independent experiments. * *p* < 0.01 *vs.* control group (one-way ANOVA followed by Tukey test).

Our first experiments were designed to evaluate possible toxic effects resulting from the exposure of primary cerebellar mixed cultures to an aqueous M1 extract of mahogany leaves. Cultures were exposed to M1 extract for 24 h (acute) and 72 h (sub-chronic) at increasing concentrations ranging from 10 ng/mL and 200 μg/mL. Our results showed that acute, but not sub-chronic exposure to M1 induced a concentration-dependent increase in cell viability, as measured by the colorimetric MTT assay (Figure 2).

As shown, sub-chronic exposure to the compound resulted in decrease in cell viability at the concentrations of 100 and 200 μg/mL, compared to control values.

### 2.4. Cytoprotective Effect of an Aqueous Extract of Mahogany Leaves against Methyl Mercury-Induced Neurotoxicity

**Figure 3:**
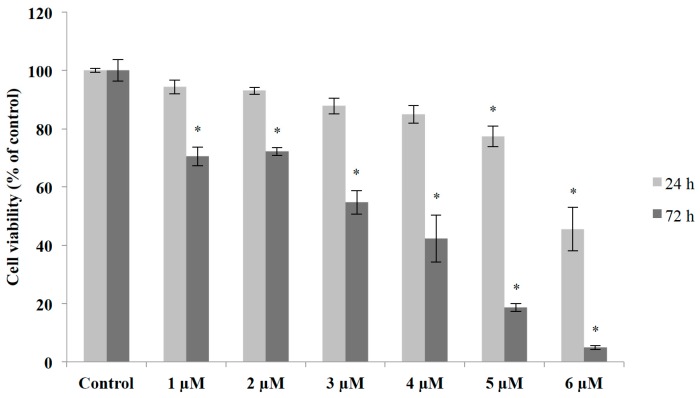
Effects of acute (24 h) and sub-chronic (72 h) exposure of methyl mercury in primary cerebellar cultures. Cell viability was determined by MTT assay and is expressed as the percentage of untreated controls; mean + S.E.M. values of three-four independent experiments. * *p* < 0.01 *vs.* control group (one-way ANOVA followed by Tukey test).

Acute (24 h) and sub-chronic (72 h) exposure of primary cerebellar cultures to methyl mercury (MeHg) resulted in decreased cell viability time- and was dose-dependent (measured by MTT assay), under our experimental conditions (Figure 3).

In order to study the cytoprotective effects of *Swietenia macrophylla*, an extract rich in phenolic and flavonoids compound, and considering oxidative stress as an important effect of mercurial compounds on nervous tissue, we performed experiments where mixed cultures were exposed concomitantly to MeHg (6 μM and 3 μM for 24- and 72-h exposure, respectively) and different concentrations of M1 aqueous extract (10, 50 and 100 μg/mL).

**Figure 4:**
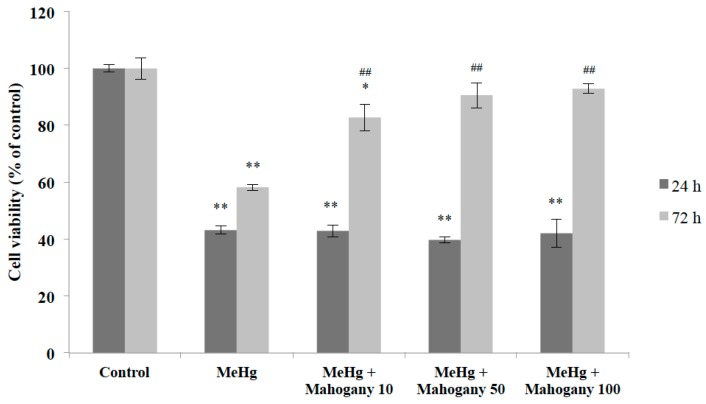
Evidence for cytoprotective effects of M1 aqueous extract on methyl mercury-induced cell loss. Cell viability was determined by MTT assay and is expressed as the percentage of untreated controls; mean + S.E.M. values of three-four independent experiments. * *p* < 0.05 and ** *p* < 0.01 *vs.* control group. **^##^** *p* < 0.01 *vs.* MeHg group (one-way ANOVA followed by Tukey test). (MeHg: methyl mercury; Mahogany 10: aqueous extract of mahogany leaves 10 μg/mL; Mahogany 50: aqueous extract of mahogany leaves 50 μg/mL; Mahogany 100: aqueous extract of mahogany leaves 100 μg/mL).

As a result, concomitant addition of M1 extract (10, 50 and 100 μg/mL) to cultures exposed to MeHg (6 μM) for 24 h resulted in no detectable effect on cell viability. On the other hand, simultaneous addition of the same concentrations of M1 extract to cultures exposed to MeHg (3 μM) for 72 h resulted in reversal or inhibition of the cytotoxic effects induced by MeHg in all concentrations tested. These results suggest that extract of *Swietenia macrophylla* provides significant protection to primary cerebellar cultures against damage caused by methyl mercury (Figure 4). Also, since this protective effect could only be observed for cultures exposed to MeHg for longer time (72 h), our data suggest that it could be related to an ability of M1 extract to promote gradual chelation against oxidative insult over time.

## 3. Experimental Section

### 3.1. Reagent and Standards

Formic acid, methanol, MTT-tetrazole (3-(4,5-dimethylthiazol-2-yl)-2,5-diphenyltetrazolium bromide), potassium chloride, poly-l-lysine, glucose, HEPES and all standards were purchased from Sigma-Aldrich (Steinheim, Germany). The water was purified with a Direct-Q^®^ 3 system (18.2 Mohm resistivity, Merck KGaA, Darmstadt, Hessen, Germany). Additionally, Dulbecco Modified Eagle Medium (DMEM), fetal bovine serum, horse serum, penicillin/streptomycin antibiotics and trypsin/EDTA (Gibco-Invitrogen, Grand Island, NY, USA) were employed.

### 3.2. Plant Material

*Swietenia macrophylla* leaves were collected at Aurora do Pará, State of Pará, Brazil. The plant was identified by a botanic expert from the Botany Department, Universidade Federal Rural da Amazônia (Belém, State of Para, Brazil) and a voucher specimen (number 1320) was deposited at the Herbarium of this institution. The leaf samples were weighed (3100 g) and taken to the laboratory immediately after harvesting. The samples were dried at room temperature packed in paper bags for a period of 30 days. After this period, a new weighing of the dried leaves (1642 g) indicated a dehydration of 47.03%.

### 3.3. Preparation of S. Macrophylla Leaf Tea

A sample of 100 g dried leaves was crushed and placed in 2.5 L of water and heated for 40 min to a temperature of 100 °C (extraction by decoction). Next, the warm mahogany tea was passed through a paper filter and the crude extract was lyophilized for 48 h to provide M1. The mass obtained was 17.09 g with a yield of 17.1%. The same mass of dried leaves was crushed and placed into 2.5 L of boiling water at a temperature of 100 °C for 5 min (extraction by infusion). The warm mahogany tea was then passed through a paper filter and the crude extract was lyophilized for 48 h to provide M2. The mass obtained was 8.82 g with a yield of 8.8%. The LC-MS sample was prepared using a 13 mm film × 0.2 μm PTFE 13-2 Iso-Disc™ membrane filter (Supelco-Sigma-Aldrich, St. Lois, MO, USA).

### 3.4. TEAC Assay

The antioxidant capacity was measured using the TEAC assay, with the radical cation ABTS^+•^ (Sigma, St. Louis, MO, USA) and K_2_S_2_O_8_ (Sigma) as the oxidant agent, as adapted by Silva *et al.* [15] from the procedure proposed by Re *et al.* [16] to be used with a microplate reader. The TEAC value represents the antioxidant reactivity relative to a standard of 1.0 mmol/L Trolox, a water-soluble synthetic derivative of vitamin E. In this study, the TEAC value was expressed as mmol of Trolox equivalent (TE) per g of dry extract or dry fraction. All analyses were done in triplicate.

### 3.5. Folin-Ciocalteau Assay

The antioxidant capacity was measured by the Folin-Ciocalteau colorimetric method [20,21]. Measurements were carried out by reaction of the Folin-Ciocalteau reagent (1 N) with Na_2_CO_3_ diluted in water (75 g/L) and samples diluted in water, during 30 min and measured at 735 nm. The assay were developed in triplicate and calculations based on a calibration curve obtained with gallic acid. The results were expressed as milligram of gallic acid equivalents (GAE) per g of dry extract or dry fraction.

### 3.6. Analytical LC-MS/MS

LC-MS analysis was performed on a XEVO G2-SQ-TOF mass spectrometer (Waters Corp., Milford, MA, USA) equipped with a lockspray source where an internal reference compound (leucine-enkephalin) was introduced simultaneously with the analyte for accurate mass measurements. Compounds were separated in a HSS C18 column (Waters Corp.; 50 mm; 2.1 mm; 1.8 μm particle size) using 0.1% aqueous formic acid (solvent A) and 0.1% formic acid in methanol (solvent B). Column temperature was maintained at 40 °C. 

Gradient elution was performed with 0.1% formic acid in ultra-pure water (solvent A) and methanol (solvent B), delivered at a flow rate of 0.5 mL/min as follows: 2% B in 1 min, 2%–95% B in 8 min, 95% B during 1 additional minute. The gradient elution was followed by a 5 min post-run at initial conditions for equilibration of the column. The injection volume for the extract was 5 μL. The analysis was done in ESI positive ion mode. A confirmation method was also developed using a MS^e^ method to confirm overlapped peaks in the extract chromatogram. For this, the same MS source parameters and UPLC conditions were applied.

Electrospray mass spectra data were recorded in a positive ionization mode for a mass range from *m*/*z* 50 to 1000 with a scan time of 0.1 s. The source temperature was set to 150 °C with a cone gas flow of 20 L/h. The desolvation gas flow was set to 600 L/h at a temperature of 250 °C. The capillary was set at 3.5 kV with cone voltage at 20 V. Collision-induced fragmentation (CID) of the analytes was achieved using 20–40 V of energy with argon as the collision gas. MassLynx software (Waters) was used for system control and data acquisition.

### 3.7. Cell Culture

Primary cerebellar cultures were obtained from 5–7 days-old Wistar rats. The Animal Care and Use Committee of the University Federal of Pará approved all of the animal protocols used. Cultures were prepared as described previously to Ahlemeyer and Baumgart-Vogt [22], with minor modifications. Briefly, brain was rapidly removed and dissected in a petri dish with sterile dissection medium (160 mM NaCl; 5.3 mM KCl; 0.44 mM KH_2_PO_4_; 0.33 mM Na_2_HPO_4_; 4 mM NaHCO_3_; 5.5 mM glucose; antibiotics and antifungal). The brain meninges were carefully removed and the cerebellum was incubated for 15 min at 37 °C in a trypsin-EDTA 0.05% solution for enzymatic digestion. The digested tissue was transferred to culture medium DMEM containing 10% fetal bovine serum, 10% horse serum, 25 mM KCl, penicillin-streptomycin 10,000 U/mL, and gently mechanically disrupted with a Pasteur pipette. After brief decantation, cells in suspension were seeded in multi-well plates previously treated with poly-l-lysine in DMEM supplemented with 10% FBS, 10% HS, 25 mM KCl and penicillin-streptomycin, and maintained at 37 °C with 5% CO_2_ in a humidified atmosphere for 8 days, at which point cultures were confluent and the experiments were performed.

### 3.8. Methyl Mercury-Induced Oxidative Damage in Primary Cerebellar Cultures

Treatment consisted in the addition of methyl mercury dissolved in water to the primary cerebellar cultures, for two different periods of exposure (acute, 24 h and sub-chronic, 72 h) in concentration ranging from 1 µM to 6 µM.

### 3.9. Treatment with Aqueous Extract of Swietenia Macrophylla to Neuroprotective Assays

The *Swietenia macrophylla* extract was submitted to a serial dilution. To check possible toxic effects, primary cerebellar cultures were treated with the extract in concentrations ranging from 10 ng/mL to 200 µg/mL (final concentration in culture medium). Cell viability was examined by MTT assay. After that, three concentrations (non-toxic) were chosen for the evaluation of the possible neuroprotective effect.

### 3.10. Analysis of Cell Viability

Cell viability was measured by the MTT (3-(4,5-dimethylthiazol-2-yl)-2,5-diphenyltetrazolium bromide) method. Each experimental condition was performed in triplicate wells (*n* = 3 to 4 independent experiments). The control-wells were considered as having 100% viability and then compared with treated-wells in that group. Data were described as standard error mean (±S.E.M.).

### 3.11. Statistical Analysis

Results are expressed as mean values (± S.E.M.). The statistical evaluation of the data was performed by One-way analysis of variance (ANOVA) followed by Tukey *post hoc* test. *p* < 0.05 was accepted as statistically significant.

## 4. Conclusions

In this study, tea from mahogany leaves showed promise as an antioxidant source, using two antioxidant reference methods. In this sample, 27 polyphenols have been identified, among them, nine phenolic acids and 18 flavonoids, particularly 3-*O*-flavonols*.* Additionally, the LC-HRMS system provided unequivocal identification of these phenolic compounds with high mass accuracy. Such compounds are usually considered responsible for antioxidant capacity. Our results also showed that concomitant exposure of the same cultures to MeHg and aqueous extract resulted in significant increase in cell viability in all doses used, suggesting that this extract, provides significant cytoprotection in the MeHg-induced *in vitro* neurotoxicity and above all, that mahogany leaves, a waste material from the timber industry, could be a useful source of bioactive compounds, adding value to this species.
